# A Fast, Reliable, and Sensitive Method for Detection and Quantification of *Listeria monocytogenes* and *Escherichia coli* O157:H7 in Ready-to-Eat Fresh-Cut Products by MPN-qPCR

**DOI:** 10.1155/2014/608296

**Published:** 2014-05-15

**Authors:** Pasquale Russo, Giuseppe Botticella, Vittorio Capozzi, Salvatore Massa, Giuseppe Spano, Luciano Beneduce

**Affiliations:** Department of Agriculture, Food and Environmental Sciences, University of Foggia, Via Napoli 25, 71122 Foggia, Italy

## Abstract

In the present work we developed a MPN quantitative real-time PCR (MPN-qPCR) method for a fast and reliable detection and quantification of *Listeria monocytogenes* and *Escherichia coli* O157:H7 in minimally processed vegetables. In order to validate the proposed technique, the results were compared with conventional MPN followed by phenotypic and biochemical assays methods. When *L. monocytogenes* and *E. coli* O157:H7 were artificially inoculated in fresh-cut vegetables, a concentration as low as 1 CFU g^−1^ could be detected in 48 hours for both pathogens. qPCR alone allowed a limit of detection of 10^1^ CFU g^−1^ after 2 hours of enrichment for *L. monocytogenes* and *E. coli* O157:H7. Since minimally processed ready-to-eat vegetables are characterized by very short shelf life, our method can potentially address the consistent reduction of time for microbial analysis, allowing a better management of quality control. Moreover, the occurrences of both pathogenic bacteria in mixed salad samples and fresh-cut melons were monitored in two production plants from the receipt of the raw materials to the early stages of shelf life. No sample was found to be contaminated by *L. monocytogenes*. One sample of raw mixed salad was found positive to an H7 enterohemorrhagic serotype.

## 1. Introduction

Ready-to-eat (RTE) fresh vegetables and fruits have become an established product in worldwide markets whose acceptance considerably increased in the last years. However, the growing popularity of these high quality fresh food products poses many safety concerns. Indeed, fresh-cut vegetables are a potential vehicle of transmission of foodborne pathogens,* Listeria monocytogenes* and* Escherichia coli* O157:H7 being among the most hazardous. Generally, infection with* L. monocytogenes* may cause mild febrile gastroenteritis, but in susceptible individuals, such as young, old, pregnant, and immune-compromised, invasive listeriosis can result in more serious diseases including meningitis, septicaemia, preterm birth, miscarriage, and rather high mortality rate [1].* E. coli* O157:H7 is an enterohemorrhagic pathogen causing bloody diarrhoea and, in the worst cases, haemolytic uremic syndrome that can result in severe illness or even death. In the last years, outbreaks caused by the consumption of fresh vegetables contaminated with* E. coli* O157:H7 [2–6] or* L. monocytogenes* [7–10] have become increasingly recognized in developed countries. From a technological point of view, fresh-cut vegetables are considered “minimally processed food” and are hence characterized by the absence of treatment to break down the microbial load, with the exception of washing with chlorinated water and the compliance of the cold chain [11]. Fresh-cut contamination can occur at pre- and postharvest levels, with environment, irrigation water, handling of the product, and food plant uncleanness among the main critical points [12]. In particular, the ability of both foodborne pathogens to adhere to food and food-contact surfaces, thus forming or colonizing preexistent biofilm, represents a threat for the public health due to the risk of cross-contamination during food processing [13, 14]. Most recently, several food surveys throughout the world reported the detection of* L. monocytogenes* and* E. coli* O157:H7 in raw RTE vegetable salads sold at market retails [15–19]. From an industrial viewpoint, the prevalence of these events is a serious hazard for fresh-cut producers because it involves economic losses due to the recall of the food from the market and damage to the company image, impacting its intangible capital. The current European regulation for fresh-cut vegetables stipulates the absence of* E. coli* O157:H7 in 25 g of food, while for* Listeria monocytogenes* a limit concentration of 100 CFU g^−1^ is tolerated at the end of the shelf life [20]. Since minimally processed vegetables are generally characterized by short shelf life (7–15 days), rapid detection and quantification of human pathogens become a major challenge for producers as well as retail traders. Enumeration of* L. monocytogenes* and* E. coli* O157:H7 in food is generally done by the most probable number (MPN) method, which requires replicated dilution series of food in selective enrichment broth followed by plating on selective agar plates and subsequent biochemical assays for species identification [21]. Although MPN method has the advantage of enabling detection of the target pathogen even when it is present in low numbers, it is laborious and requires several days for confirmation of results. Therefore, in the last years, a considerable number of detection methods using faster molecular tools, mainly based on PCR techniques, have been proposed [16, 17, 21–23]. Currently, several genes have been suggested as targets for the molecular detection of* L. monocytogenes* [16, 24, 25] and* E. coli* O157:H7 [26]. The most recent advances in PCR methods are focused on the reduction of the limits of detection and quantification [27, 28], on the discrimination between dead and live cells [22] or the simultaneous detection of different foodborne pathogens [22, 27–29]. The performances of these methods were, in some cases, successfully compared with the official protocols or with diagnostic commercial kits [25, 28].

In the present work we integrated conventional MPN technique with qPCR, in order to preserve or explore the advantages of both methods, sensitivity of MPN, and reliability and quickness of qPCR. Moreover, we developed a qPCR enrichment-based method for a fast and reliable detection of* L. monocytogenes* and* E. coli* O157:H7 in minimally processed vegetables. In order to validate the proposed technique, the results were compared with conventional culture-dependent methods.

## 2. Materials and Methods

### 2.1. Bacterial Strains and Growth Conditions

*Listeria monocytogenes* CECT 4031 and* Escherichia coli* O157:H7 CECT 4267 were purchased from the Spanish Type Culture Collection (CECT, Valencia, Spain). The non-*monocytogenes Listeria* spp. strains,* Listeria ivanovii* IZSP B45, and* Listeria innocua* IZSP B48 were kindly provided by Istituto Zooprofilattico Sperimentale di Puglia e Basilicata (IZPS, Foggia, Italy). Non-STEC* Escherichia coli* DSM 3423 was purchased from the German Collection of Microorganisms (DSMZ, Braunschweig, Germany). All strains were routinely cultured at 37°C in TSB broth (Oxoid, Hampshire, UK) until reaching mid exponential phase.* Lactobacillus plantarum* WCFS1 was used as additional negative control and grown at 30°C on MRS broth (Oxoid).

### 2.2. Food Samples and Artificial Contamination

Minimally processed fresh-cut mix salads (lettuce, radicchio, and endive) were randomly purchased at local markets in Foggia (Italy) and stored at 4°C for a maximum of 24 h prior to analyses. All samples were investigated for the presence of* Listeria* spp. and* E. coli* O157:H7 as recommended by the ISO protocol 11290-1 from International Organization for Standardization [30]. All samples were negative for the presence of both pathogens and were used for subsequent artificial contamination experiments.

For artificial inoculation,* L. monocytogenes* CECT 4031 or* E. coli* O157:H7 CECT 4267 grown at middle exponential phase were added to the corresponding enrichment selective media used for the MPN assays (see below) in order to obtain a contamination level ranging from 0 to 3 Log CFU g^−1^ of sample. For the qPCR detection without selective enrichment, salads samples were also inoculated at a level of 4 and 5 Log CFU g^−1^.

### 2.3. MPN Enumeration of* Listeria monocytogenes*

Salad samples (25 g) were added to 225 mL of Fraser Broth (Oxoid) supplemented with Fraser Selective Supplement SRO156E (Oxoid) and homogenized in a stomacher (BagFilter, Interscience, FR) for 2 min. Triplicate series of tubes containing decimal serial dilution from 10 to 10^−5^ grams of homogenate were incubated for 48 hours at 37°C in the same media. After incubation, aliquots of enrichment broth were taken from dark tubes (containing presumptive* Listeria* spp.) and streaked onto Oxford agar plates (Oxoid) and PALCAM agar (Oxoid). Plates were incubated at 37°C for 48 h and five typical colonies were picked for purification on TSA + 0.6% yeast extract plates (Oxoid). Then, plates were incubated for 24 h at 37°C and Gram-positive, catalase positive colonies were streaked on blood agar (37°C, 24 h). Hemolytic colonies were identified as* L. monocytogenes* using API Listeria strips (Biomerieux, Marcy l'Etoile, FR). The MPN value and 95% confidence intervals were determined by the number of positive tubes obtained in serial dilutions as reported by the USDA guidelines [31].

### 2.4. MPN Enumeration of* Escherichia coli* O157:H7

Salad samples (25 g) were added to 225 mL TSB (Oxoid) supplemented with 20 mg L^−1^ of novobiocin (Sigma, MO, US) and 1.12 g L^−1^ of bile salts (Oxoid) as reported by Fusco et al. [32] and homogenized in a stomacher (Bag Mixer, Interscience) for 2 min. Triplicate series of tubes containing decimal serial dilution (from 10 to 10^−5^ grams of homogenate) were incubated for 48 h at 37°C. Turbid cultures were considered to be presumptive positive. Confirmation of presumptive* E. coli* O157:H7 was carried out by spread-plating 100 *μ*L samples from each turbid tube onto tellurite-cefixime Sorbitol MacConkey agar plates (Becton Dickinson, Sparks, MD) supplemented with 200 *μ*g mL^−1^ ampicillin. Plates were incubated overnight at 37°C and colonies showing the typical morphology (colorless or neutral/gray with a smoky center and 1-2 mm in diameter) were considered positive. For further confirmation, a portion of each typical colony was picked and tested using RIM* E. coli* O157:H7 latex agglutination assay (Remel Inc., Lenexa, KS).

### 2.5. DNA Extraction

Genomic DNA was extracted from dilution tubes considered for MPN enumeration by comparing two methods: the DNeasy Blood and Tissue kit (Qiagen, Milano, IT) according to the manufacturer's instructions and the boiling method reported by de Oliveira et al. [16].

DNA was also extracted from artificially inoculated salads samples, after homogenization in the enrichment broth, after 0, 2, 4, 6, and 24 hours, respectively. Briefly, 10 mL of the homogenate were centrifuged for 10 min at 5.000 g. Supernatant was then discarded and DNA extracted from cell pellet using the DNeasy Blood and Tissue kit (Qiagen) following manufacturer's instructions for Gram-positive or Gram-negative bacteria depending on the assayed microorganism.

DNA concentration was measured using a BioTek Eon spectrophotometer (BioTek, VT, USA) and its integrity checked by visualization on 1.2% agarose gels. Then, samples were stored at −20°C before analyses.

### 2.6. Real-Time PCR Conditions

*L. monocytogenes* specific primers and hybridization probe tagged with FAM fluorescent dye as designed by Rodríguez-Lázaro et al. [24] were used to amplify a 64-base pair fragment of the listeriolysin O gene (*hlyA*) (Table 1).* E. coli* O157:H7 was detected by using primers and probes targeting* fliC* H7 (encoding the flagellar antigen H7) and* rfbE* (coding for the antigen O157) genes [26], tagged with FAM and VIC fluorescent dye, respectively (Table 1). Primers and probes were synthesized by PRIMM Biotech (Milano, IT). qPCR assays were performed on an AB 7300 Real-Time PCR System (Life Technologies, Monza, IT). Amplification was carried out in a final volume of 20 *μ*L including 3 *μ*L of template DNA, 10 *μ*L iTaq Universal probes Supermix (Bio-Rad, Milano, IT), 0.1 *μ*M of each primer, and 200 nM of probe. Amplification conditions for* L. monocytogenes* detection were as follows: initial denaturation at 95°C for 10 min followed by 45 cycles of 15 s at 95°C and 1 min at 63°C. Cycling program for* E. coli* O157:H7 detection was as follows: 95°C for 10 min followed by 45 cycles of 10 s at 95°C and 30 s at 60°C. The specificity of the assay was tested by using DNA templates from pure cultures of* E. coli* O157:H7,* L. monocytogenes*, non-*monocytogenes Listeria* spp., non-STEC* E. coli*, and* L. plantarum*. Quantification was performed by interpolating values from unknown samples in a standard regression curve generated from tenfold dilutions of triplicate samples at known DNA concentration of the respective microorganisms. Real-time PCR assays were performed in duplicate and included a negative control and no template control in each run. Data were expressed as the mean of three independent experiments.

Amplifications of the* uidA* gene were carried out by qPCR following the methods reported by Ram et al. [33]. Amplification of the* stx*2 gene was carried out following the method of Jinneman et al. [34]. Reaction total volumes were 20 *μ*L; 3 *μ*L of template DNA were added, to a real-time PCR mix containing Power SYBR Green PCR Master Mix (Applied Biosystems, Foster City, CA) and 100 nM of each primer according to the manufacturer's instructions. Cycling program was as follows: for* uidA*, initial denaturation at 95°C for 3 min followed by 45 cycles of 20 s at 95°C, 30 s at 54.5°C, and 30 s at 72°C. Cycling program for* stx2* detection was as follows: 95°C for 10 min followed by 45 cycles of 10 s at 95°C and 30 s at 60°C.

### 2.7. Application of the qPCR Method to RTE Food Production Chain

Minimally processed fresh-cut samples representative of 2 production batches of rocket, mixed salad, and* piel de sapo* melons, respectively, were provided by two different fresh-cut vegetables and fruit companies. For each food, samples were analyzed by MPN-qPCR and culture-dependent methods at three different stages: raw, after processing, and at three days of shelf life at 4-5°C. All samples were used for demonstration activity only and not purchased by the companies involved.

## 3. Results

### 3.1. Optimizing* Listeria monocytogenes* qPCR Assay Conditions

In the present study, the qPCR method developed by Rodríguez-Lázaro et al. [24] was adapted to our purposes and employed in order to detect and quantify* L. monocytogenes* in artificially contaminated fresh-cut vegetables. Specificity of the assay was confirmed in all preliminary assays (data not shown), performed using 1 ng of genomic DNA from* Listeria monocytogenes* and non-*monocytogenes* strains as template.

**Figure 1:**
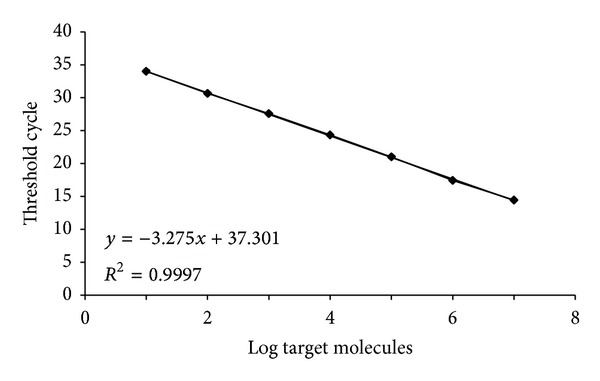
Standard curve and amplification plot of the 64 bp* hlyA* gene fragment generated by qPCR amplification of serially diluted purified DNA of* Listeria monocytogenes* represented as log of genome equivalents/reaction. Trend line equation and the corresponding square regression coefficient (*R* ^2^) are shown.

Detection and quantification limits of the qPCR assay were investigated by using DNA extracted from overnight cultures of* Listeria monocytogenes* CECT 4031 strain. Serial dilutions of DNA were subjected to qPCR and a standard curve was constructed by using* hlyA* as target gene. Amplification profile of serial dilutions and standard curve are shown in Figure 1.

Amplification reactions were carried out with a range of DNA concentrations approximately corresponding to 1 × 10^7^–1 × 10^0^ target molecules. With the target sequence being part of a single copy gene, number of target molecules was estimated as total DNA/mass of a single* L. monocytogenes* genome *≈*2.94 × 10^−15^ g. The standard curve showed a linear relationship, spanning 7 logs, between log input DNA and threshold cycle. The slope of the curve was −3.27, close to the theoretical optimum (−3.32), and corresponding to 102% amplification efficiency, calculated by the formula (Efficiency = 10^−1/slope^ − 1). Square regression coefficient was *R* ^2^ = 0.999 indicating that the qPCR assay is highly linear in the considered range. There was no overlapping of confidence intervals based on standard deviation of *C*_*T*_ values down to 1 × 10^1^ target molecules indicating that reliable quantification is possible to this limit. The LOD was found to be lower, corresponding to 5 target molecules (mean *C*_*T*_ value: 37.8 and st. dev.: 3.7). This data led us to consider this qPCR assay suitable for development of MPN-qPCR and enrichment free detection protocols.

### 3.2. *Escherichia coli* O157:H7 qPCR Assay

We adapted the original protocol by Perelle et al. [26] for use in a duplex reaction but eventually found that, despite the duplex assay based on these primers and probe guaranteed high specificity, single reactions were more sensitive than duplex. Sensitivity was determined as limit of detection, namely, the lowest concentration at which 95% of the positive samples are detected. Duplex reaction showed a LOD that was about one order of magnitude higher than the LOD of single reactions (data not shown).

**Figure 2:**
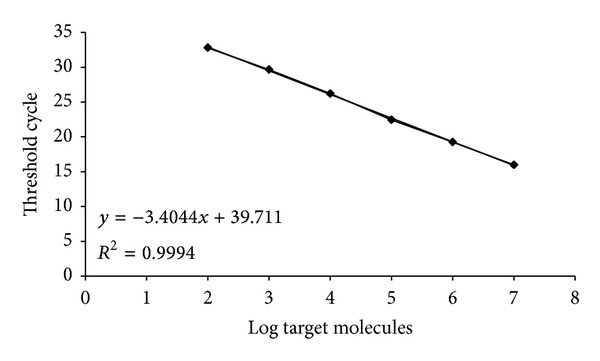
Standard curve and amplification plot of the 96 bp* fliC* gene fragment by qPCR amplification of serially diluted purified DNA of* Escherichia coli* O157:H7 represented as log of genome equivalents/reaction. Trend line equation and the corresponding square regression coefficient (*R* ^2^) are shown.

Therefore, we detected and quantified* E. coli* O157:H7 by adopting single reactions, performed at the same time and in the same experiment plate. Amplification reactions were carried out with a range of DNA concentrations approximately corresponding to 1.0 × 10^7^–1.0 × 10^0^ target molecules (single* E. coli* genome mass *≈*6.13 × 10^−15^ g), and two standard curves, one for each primer/probe system, were constructed. From now on in our exposition we will make reference to the less performing primer-probe system, targeting gene* fliC* H7, as it determines the detection limit of the assay. Standard curve and amplification plot for* fliC* H7 assay are shown in Figure 2.

The standard curve showed a linear relationship (spanning 6 logs) between log input DNA and threshold cycle (Figure 2). The slope of the curve was −3.40 and the square regression coefficient was *R* ^2^ = 0.999. Based on these data, efficiency of the assay is 96.84%. The estimated limit of quantitation (LOQ) is 1 × 10^2^ target molecules. The estimated detection limit (LOD) was one order of magnitude lower, corresponding to 10 target molecules (mean *C*_*T*_ value was 37.16 and st. dev. was 0.78).

### 3.3. *L. monocytogenes* and* E. coli* O157:H7 Enumeration by MPN-qPCR

In our experiments we used a MPN protocol integrated with qPCR in which presumptive positive MPN tubes are directly checked by qPCR for confirmation. In order to evaluate the reliability of our method on fresh-cut vegetables we applied our protocols on artificially inoculated salads (0 to 3 Log CFU g^−1^) with both pathogens. Salad samples not inoculated and previously assayed for both the target pathogens were used as negative control.

Enumeration of* Listeria monocytogenes* by the MPN method from artificially contaminated foods was performed after 48 h of incubation of triplicate serial dilutions in selective Fraser Broth. Aesculin hydrolysis in the positive tubes resulting in turning of culture medium to black allowed the definition of the three consecutive dilutions to determine the initial contamination by referring to the corresponding MPN tables [31]. Presumptive* L. monocytogenes* positive tubes were phenotypically and biochemically confirmed in all cases. Moreover, no typical* L. monocytogenes* colonies were observed on selective media when negative tubes from the same dilutions were assayed. Black tubes from negative controls were never observed by MPN. This result was also confirmed by conventional techniques (Table 2).

*E. coli* O157:H7 MPN enumeration was determined considering positive the tubes showing turbidity after 48 h incubation in TSB supplemented with novobiocin and bile salts and further confirmed for the ability to grow on CT-SMAC. Differently from what is observed for* L. monocytogenes*, MPN enumeration of* E. coli* O157:H7 gave some presumptive positive results in control and in artificially contaminated samples (Table 2) that were not confirmed by conventional methods. Interestingly, although an incubation time of 48 h was used, bacterial growth was observed in all the positive tubes used for the MPN enumeration already after 24 h of enrichment. Although the DNA yield was higher when the commercial kit was used (data not shown), no discrepant results were observed using as template the DNA obtained from both the kit and the boiling method. Indeed, the amplification of* hlyA*,* fliC* H7, and* rfbE* genes by qPCR always confirmed the presence of pathogens only in the cultures positive to the conventional approaches while no signal was detected submitting DNA from negative tubes to the molecular analysis (Table 2).

### 3.4. Detection of* L. monocytogenes* and* E. coli* O157:H7 by qPCR

With the aim to further reduce the time for the detection of both pathogens we assayed a qPCR approach not associated with MPN enumeration. For the evaluation of LOQ and LOD of the qPCR methods without MPN step, artificially contaminated fresh-cut vegetables used for MPN enumeration were incubated at 37°C and samples analyzed by qPCR after 0, 2, 4, 6, and 24 h of enrichment, respectively. As previously reported for vegetables matrices [35], DNA was extracted from 10 mL of homogenate by using the DNeasy Blood and Tissue kit (Qiagen).* L. monocytogenes* was detectable at contamination levels as low as 10 CFU g^−1^ with two hours of enrichment (mean *C*_*T*_ value was 36.73 and st. dev. was 1.12). Limit of detection for* E. coli* O157:H7 was 10 CFU g^−1^, after two hours of enrichment (mean *C*_*T*_ value was 37.11 and st. dev. was 2.48). Limit of quantitation (LOQ) was 10^4^ CFU g^−1^ without selective enrichment for both pathogens (data not shown).

### 3.5. Screening of RTE Samples

Samples of ready-to-eat vegetables and fruits provided by two different companies were investigated for the presence of* Listeria monocytogenes* and* E. coli* O157:H7 by using both MPN-qPCR and conventional culture-dependent methods. Black tubes were not observed by MPN enumeration of all the samples, suggesting the absence of presumptive* Listeria* spp. Indeed,* L. monocytogenes* was never detected by qPCR nor conventional methods (data not shown). Compared to* L. monocytogenes* assays,* E. coli* O157:H7 gave some presumptive positive results after the enrichment step, but in no case they were confirmed by culture methods (Table 3). The amplification of* rfbE* gene by qPCR did not give positive signals in any sample. Interestingly, we found that one sample of mixed raw salad (prior to processing) was positive by using* flic* H7 as target gene (Table 3). For further investigation the sample was processed by qPCR targeting* uidA* (beta-glucuronidase gene) and* stx2* (shiga toxin 2 gene, shared by all enterohemorrhagic serotypes) and found to be positive for both (data not shown). Basing on the* uidA* and* stx2* assays, the sample was found to be contaminated by a strain of potentially pathogenic H7 serotype, other than O157, even though it was not possible to isolate the H7 serotype on culture media.

## 4. Discussion

Fresh-cut packaged fruits and vegetables sold in the market are generally considered a product of high quality and freshness. However, they may represent an underestimated public health risk due to the potential presence of pathogenic bacteria, like* Listeria monocytogenes* and* Escherichia coli* O157:H7 [36]. The existing regulations in Europe stipulate that the identification of these foodborne pathogens is carried out by culture-dependent methods. Nonetheless, an inexpensive analysis and a fast preliminary result based on a molecular approach could be remarkable for fresh-cut producers to integrate the conventional methods and thus allowing a promptly intervention on presumptive contaminated products and a more drastic sanitization of the plant. Therefore, a rapid detection of the pathogens should improve the internal quality control assessment ensuring a greater safety to the consumer.

In the last years, PCR-based techniques have been the subject of considerable focus and ISO guidelines have been established for the detection of foodborne pathogens (ISO 22174:2005, ISO/TS 20836:2005, ISO 20837:2006, and ISO 20838:2006). Particularly, real-time quantitative PCR is considered a method of choice for the detection and quantification of microorganisms [37].

However, a methodology can be considered attractive to implement routine checks of commercial products only if sufficiently simple, fast, efficient, and relatively inexpensive. In order to reduce the time for the analysis and the corresponding cost, some recent works addressed the simultaneous detection by qPCR of pathogenic bacteria including* L. monocytogenes* and* E. coli* O157:H7 [22, 27–29, 35]. Nonetheless, main efforts have been made to improve critical points such as the sensitivity and specificity in order to ensure a fast and reliable method.

Although, several works reported on a rapid detection method for* L. monocytogenes* in food, in few cases the studies focused on its quantification from vegetables matrices by coupling MPN with PCR [17] or qPCR [16, 21] after an enrichment of 48 h. Similarly, MPN-PCR has been successfully used to detect* E. coli* O157:H7 in milk and dairy products [32, 38], but to our knowledge no enumeration effort has been carried out on fresh and RTE vegetables.

Therefore, in this study we aimed at the foodborne pathogens quantification by integrating MPN with qPCR, in order to considerably reduce the time required for confirmation by conventional phenotypic and biochemical assays. The first goal was the comparison of the results obtained by MPN-qPCR with those ones from culture-dependent methods. Our method enabled the detection and—indirectly—quantification of* L. monocytogenes* and* E. coli* O157:H7 when inoculated a concentration of as low as 1 CFU g^−1^, after 24 hours of incubation in the corresponding selective media, with a gain of about 4 days compared to the standard culture method. It has to be remarked that in real conditions the pathogen cells on vegetable surfaces are submitted to several stresses such as washing with chlorinated water, low temperatures, and microbial competition, thus resulting in a slower recovery and growth rate. For this reason, an increase of the incubation time to 48 h is anyway recommended for MPN enumeration when real samples are analyzed. Only for* E. coli* O157:H7 false positive MPN results after the selective enrichment were recorded. The aspecific turbidity of MPN tubes from control and artificially contaminated samples was frequent above the theoretical inoculum of 10^2^ CFU g^−1^. We argue that Fraser is a selective medium with higher inhibition of non-*Listeria* spp., while resistance to selective agents such as bile salts and novobiocin is a more widely distributed feature between enteric bacteria, thus resulting in the growth of bacteria and/or* E. coli* strains different from O157:H7.

With the aim to further reduce the time for the detection of both pathogens we assayed a qPCR approach not associated with MPN enumeration by using as target DNA extracted after 0, 2, 4, and 6 h of enrichment in the corresponding selective media. We observed that after 2 h it was possible to detect* L. monocytogenes* and* E. coli* O157:H7 with a limit of 10 CFU g^−1^. Although quantitative PCR methods are increasingly being used to detect bacterial pathogens in food, detection limits rarely exceed 10^2^–10^3^ CFU g^−1^ [35]. More recently, a method based on the recovery and concentration allowed the simultaneous detection and quantification of 10^2^ CFU g^−1^ for* L. monocytogenes* and* E. coli* O157:H7 in parsley and salad [28]. However, detection values below 10 CFU mL^−1^ were only achieved after selective enrichment step which required approximately 30 hours [16, 25, 29, 39]. In contrast, conventional methods require five days for determination of a negative result for* L. monocytogenes* contamination while, if a positive test result occurs, additional days are required for biochemical tests to identify the species [40].

The robustness of the results obtained by qPCR approach is closely related to the efficient recovery of bacterial DNA. Furthermore, DNA quality is critical because the efficiency of PCR amplification can be reduced by inhibitors from the matrix. In the last years, several works compared different microbial DNA extraction techniques in order to optimize yield, time, and cost of the sample preparation process depending on the food [35, 41–43]. The commercial DNeasy Blood and Tissue kit has been reported as an efficient DNA purification method from vegetable matrices [27, 35]. Thus, in this study we used the same protocol to extract the DNA during the enrichment qPCR assays.

When we used the previously described boiling method for DNA extraction [16, 17], we found that, for mixed salad samples, the results of qPCR analyses were the same as that for DNA extracted with commercial kit. This result was possible since our method is based on DNA isolation from positive MPN tubes, in which vegetable debris is codiluted with bacterial cells, thus reducing the possible PCR inhibitors present in leafy vegetables. Therefore, with the aim to propose a method for routine analysis, we suggest the last as a suitable protocol in terms of cost, times for analysis, and handling.

Several authors reported the detection of* L. monocytogenes* and* E. coli* O157:H7 in fresh-cut vegetables and fruits sold at retail markets [18, 44, 45], but the occurrence of these foodborne pathogens during food processing has not been sufficiently investigated. Fresh-cut contaminations can occur at pre- and postharvest levels [12], and often an increase of* L. monocytogenes* concentrations in fresh-cut vegetables stored under different conditions has been observed [46–48]. Therefore, our method was applied for the detection of* L. monocytogenes* and O157:H7 to RTE food production chain, by analyzing 3 different products at 3 stages of processing (see Section 2). Rocket and mix salads were selected due to their worldwide spread on the market and fresh-cut melons, since contaminated cantaloupes were involved in the most important multistate listeriosis outbreak of the last years [7].

Our results showed that MPN-qPCR always matched the outcomes of the conventional methods supporting that it is a reliable approach to discriminate both positive and negative presumptive results from MPN enumeration.

This approach could be interesting for industrial purposes since enumeration of the pathogenic microorganisms can provide an estimation of the efficacy of sanitizers treatment and represent an alarm bell to reduce the risk of cross-contaminations in the plant.

Therefore, we believe that this work will contribute to confirming the effectiveness of molecular methods as a powerful tool to complement conventional methods for a rapid detection of relevant foodborne pathogens in the fresh-cut products.

## Figures and Tables

**Table 1:** Primers and probes used in qPCR assays.

Target gene		Sequence (5′-3′)	Fragment size	Reference
*hlyA *	Forward primer	CATGGCACCACCAGCATCT	64 bp	
	Reverse primer	ATCCGCGTGTTTCTTTTCGA		[24]
	Probe	FAM-CGCCTGCAAGTCCTAAGACGCCA-TAMRA		

*rfbE *	Forward primer	TTTCACACTTATTGGATGGTCTCAA	88 bp	
	Reverse primer	CGATGAGTTTATCTGCAAGGTGAT		[26]
	Probe	VIC-AGGACCGCAGAGGAAAGAGAGGAATTAAGG-TAMRA		

*fliC *H7	Forward primer	CCACGACAGGTCTTTATGATCTGA	96 bp	
	Reverse primer	CAACTGTGACTTTATCGCCATTCC		[26]
	Probe	FAM-CCGAAAATACCTTGTTAACTACCGATGCTGC-BHQ		

**Table 2:** Quantification of *L. monocytogenes* and *E. coli* O157:H7 in artificially inoculated fresh-cut salads by MPN and confirmation of positive tubes by conventional methods and qPCR.

	Theoretical inoculum (CFU g^−1^)	3-tube dilution^1^ (mL homogenized)	Positive tubes (MPN)	True positive^2^	MPN (g^−1^)^3^ (95% c.i.)	Positive (qPCR)
*L. monocytogenes *	Control	10/1/0.1	0/0/0	n.d.	n.d.	n.d.
	1	10/1/0.1	3/2/0	3/2/0	0.93 (0.23–3.80)	3/2/0
	10	1/0.1/0.01	3/1/0	3/1/0	4.3 (0.90–18)	3/1/0
	100	0.1/0.01/0.001	3/0/2	3/0/2	64 (17–180)	3/0/2
	1000	0.01/0.001/0.001	3/1/1	3/1/1	750 (170–2000)	3/1/1

*E. coli* O157:H7	Control	10/1/0.1	3/3/3	n.d.	n.d.	n.d.
	1	10/1/0.1	3/3/3	3/1/2	>11.0	3/1/2
	10	1/0.1/0.01	3/3/1	3/2/0	46 (9–200)	3/2/0
	100	0.1/0.01/0.001	3/2/1	3/1/1	150 (37–420)	3/1/1
	1000	0.01/0.001/0.001	3/2/0	3/2/0	930 (180–4200)	3/2/0

^1^The reported dilution is referred to as the set of tubes considered for MPN enumeration.

^2^Confirmed by biochemical and immunological methods.

^3^Calculated on the basis of the true positive samples.

**Table 3:** Enumeration of *E. coli* O157:H7 in samples of rocket, mix salad and *piel de sapo* melons raw, processed, and at 3 days of shelf life by the MPN-qPCR method and using *fliC* H7 and *rfbE* as target genes.

	Sample	3-tube dilution (mL homogenized)	Positive tubes (MPN)	Positive (conventional method)	Positive *fliC* H7 (qPCR)	Positive *rfbE* (qPCR)	MPN-qPCR
Rocket	Raw	0.1/0.01/0.001	1/1/0	n.d.	0/0/0	0/0/0	n.d.
	Processed	1/0.1/0.01	2/2/0	n.d.	0/0/0	0/0/0	n.d.
	3-day shelf life	1/0.1/0.01	1/3/0	n.d.	0/0/0	0/0/0	n.d.

Mix salad	Raw	0.1/0.01/0.001	0/3/0	n.d.	0/1/0	0/0/0	+/−^1^
	Processed	1/0.1/0.01	2/3/0	n.d.	0/0/0	0/0/0	n.d.
	3-day shelf life	1/0.1/0.01	3/2/0	n.d.	0/0/0	0/0/0	n.d.

*Piel de sapo *melons	Raw	1/0.1/0.01	1/0/0	n.d.	0/0/0	0/0/0	n.d.
	Processed	10/1/0.1	1/1/0	n.d.	0/0/0	0/0/0	n.d.
	3-day shelf life	10/1/0.1	1/2/0	n.d.	0/0/0	0/0/0	n.d.

^1^+ for *fliC* (H7) gene and negative for *rfb* (O157) gene.
